# Selection in working memory is resource-demanding: Concurrent task effects on the retro-cue effect

**DOI:** 10.3758/s13414-020-02239-0

**Published:** 2021-02-19

**Authors:** Yin-ting Lin, Edyta Sasin, Daryl Fougnie

**Affiliations:** Department of Psychology, New York University Abu Dhabi, Saadiyat Island, Abu Dhabi, United Arab Emirates

**Keywords:** Visual working memory, Attention, Dual-task performance

## Abstract

In a retro-cue paradigm, after memorizing a set of objects, people are cued to remember only a subset. Improved memory from the retro-cue suggests that selection processes can benefit items stored in working memory. Does selection in working memory require attention? If so, an attention-demanding task should disrupt retro-cue effects. Studies using a dual-task paradigm have found mixed results, with only one study (Janczyk & Berryhill, Attention, Perception, and Psychophysics, 76 (3), 715–724, 2014) showing a decreased retro-cue effect by a secondary task. Here we explore a potential issue in that study – the temporal overlap of the secondary task response with the memory test presentation. This raises questions about whether the secondary task was impairing selection processes in memory or was impacting the memory response. We replicated their paradigm by inserting a tone discrimination task at the retro-cue offset, but we also included a condition in which the tone task and the memory test were temporally separated. In Experiment 1, performing the tone task did not impair the retro-cue effect. In Experiment 2, we added an articulatory suppression task as in Janczyk and Berryhill’s study, and we found that the requirement to execute the tone task impaired retro-cue effects. This impairment was independent of whether the tone and memory tasks overlapped. These findings suggest that internal prioritization can be impaired by dual-task interference, but may only occur when such interference is robust enough, for example, due to switching between multiple tasks.

## Introduction

Attention can be directed externally to select relevant sensory input while filtering out input that is irrelevant for the current goals. Previous studies have shown that using pre-cues to draw attention to relevant locations in perceptual space enhances subsequent processing and encoding of visual stimuli at those locations (Griffin & Nobre, 2003; Posner, 1980; Schmidt, Vogel, Woodman, & Luck, 2002). Interestingly, selection also occurs in working memory (WM), the short-term storage of information no longer available to the senses. An example of this comes from studies using the retro-cue paradigm (Griffin & Nobre, 2003; Landman, Spekreijse, & Lamme, 2003). In this paradigm, participants encode a set of visual objects, and next, they are instructed by the cue for which of these objects should be remembered. Retro-cueing an item improves memory performance for that item compared to the condition in which all items need to be remembered. This benefit is known as the retro-cue effect.

That both perception and working memory involve beneficial aspects of selection with similar properties has led to the idea that there is a common underlying mechanism for both forms of selection (Chun, Golomb, & Turk-Browne, 2011; Gazzaley & Nobre, 2012; Kiyonaga & Egner, 2013; see also Janczyk & Reuss, 2016; Tanoue & Berryhill, 2012). In this view, the ability to give preferential access and storage to selected items in memory would be due to shifts of attention (similar to the way attention shifts perception). Alternatively, it may be that the selection processes that create prioritization in memory do not overlap with attention or may be automatic processes. Certainly, attention is not a unitary process, and here we ask how the purposeful control of attention in perception relates to that in memory.

One way to examine this is to employ a dual-task paradigm. If the retro-cue effect arises from attentional processes, then performing an attention-demanding task during a retro-cue paradigm should impair retro-cue effects. However, studies that have utilized a dual-task approach to study whether attentional resources are involved in the retro-cue effect have received mixed results. Several studies have shown that the retro-cue effect is resistant to interference from a secondary task such as a visual search task (Hollingworth & Maxcey-Richard, 2013), a color classification task (Rerko, Souza, & Oberauer, 2014), or a digit classification task (Makovski & Pertzov, 2015). These studies provided evidence that the retro-cue effect does not require sustained attention. Also, the retro-cue effect is not affected by visual masks presented after the cue (Barth & Schneider, 2018; Makovski & Jiang, 2007; Schneider, Barth, Getzmann, & Wascher, 2017; van Moorselaar, Gunseli, Theeuwes, & Olivers, 2015). These findings might suggest that the attentional processes that give rise to the retro-cue effect are automatic and are thus not subjected to interference from another attention-demanding task or processing.

On the other hand, other studies showed that the retro-cue effect requires some time to fully develop (Pertzov, Bays, Joseph, & Husain, 2013; Souza, Rerko, & Oberauer, 2014, 2016; Tanoue & Berryhill, 2012; Wallis, Stokes, Cousijn, Woolrich, & Nobre, 2015), which might lead to the conclusion that this effect is not fully automatic. Thus, it is possible that the retro-cue effect suffers from concurrent task interference from an attention-demanding task only when such interference occurs at some critical time. This issue was raised in a study by Janczyk and Berryhill (2014). The authors investigated whether reorienting towards the retro-cued item requires attention. They argued that previous studies observed a lack of evidence for reduced retro-cue effects under dual-task conditions because they imposed too long a delay (ranging from 500 ms to 900 ms) between retro-cue offset and the secondary task (Hollingworth & Maxcey-Richard, 2013; Makovski & Pertzov, 2015; Rerko et al., 2014). They hypothesized that central attention might only be necessary around the time of cue onset, cue encoding, and briefly after that. However, subsequently, attention can be shifted to the secondary task without cost for the retro-cue effect. To test their hypothesis, Janczyk and Berryhill (2014, Experiment 1) presented a tone discrimination task during the retention interval (either 150 ms before retro-cue onset or at the retro-cue offset) of a color change-detection task. The tone task that they used is specifically thought to disrupt central attention, a form of purposeful attention thought to be involved in memory consolidation (Stevanovski & Jolicoeur, 2007) and response selection (Pashler, 1994). They found that dual-task demands impaired both WM performance and the retro-cue effect.

Janczyk and Berryhill (2014) provided evidence for a reduced retro-cue effect when the secondary task occurs close to cue onset and cue encoding, suggesting that there is a critical time window when attention is required. However, before trusting these findings, there is a potential issue with their methodology that raises questions about how to interpret the findings. The task required participants to first make a speeded response to the tone and then to respond to the memory task. Notably, the delay between the tone task and memory probe was short (400 ms or 650 ms) relative to the tone task response time (RT; the mean RT was 1,333 ms, averaged across early- and late-tone conditions, from Experiment 1 in Janczyk & Berryhill). This suggests that participants were more often than not still responding to the tone task when the probe appeared. Thus, it is far from clear that the design isolated the impact of the tone task on the retro-cue effect. Instead, responding to the tone task might have produced a bottleneck during which evidence accumulation or shifts of attention during the memory response could not proceed, reducing the effects of cue validity (Brisson & Jolicœur, 2007; Craik, Eftekhari, & Binns, 2018; Dell’Acqua, Sessa, Jolicœur, & Robitaille, 2006). Alternatively, perhaps having to engage in simultaneous tone and memory discriminations does not prevent the prioritization of information but does prevent that information from being accessed or retrieved until the tone task is completed (Carrier & Pashler, 1995; Magen, 2017; Oberauer, 2018). In addition, performing an attention-demanding task could make memory more vulnerable to retroactive interference from the memory probe (Wang, Theeuwes, & Olivers, 2018). Thus, it is possible that the results obtained by Janczyk and Berryhill stemmed from the specific design in which the tone task and the presentation of the memory probe overlapped.

Despite this issue of interpretation, the work by Janczyk and Berryhill (2014) is important as it is the singular study that found a cost of attention on the retro-cue effect. The cost was reflected by the reduction of the retro-cue effect when the cue was followed by the secondary task compared to when no secondary task was required**.** Further, their study is the only study that had a tight overlap of the retro-cue and the attention-demanding task, raising the possibility that costs only arise when the processes are close in time. The current study aimed to address the issues of interpretation in their findings. We used a paradigm very similar to theirs, but crucially we added a condition in which execution of the tone task and presentation of the memory probe do not overlap with each other. By adding this condition, we could test whether the decrement of the retro-cue effect that they observed was indeed the result of dual-task interference at the time of cue encoding. The lack of reduction of retro-cue effects under the condition when these two tasks do not overlap would suggest that previous results stemmed from the requirement to execute a tone task when the memory probe was already presented. Such findings would speak against Janczyk and Berryhill’s conclusion that attention is required to monitor processes that give rise to retro-cue effects and would open possibilities for other interpretations for their findings. This is important because no previous study has explored the role of response-related interference on the attentional interference of retro-cue effects.

## Experiment 1

### Method

#### Participants

We chose a sample size of 36 participants, the same sample size used by Janczyk and Berryhill (2014). Thirty-six New York University Abu Dhabi students and staff participated for course credit or subsistence of 50 AED per hour (18 females; mean age = 23.14 years; age range = 18–35 years). All participants reported normal or corrected-to-normal vision and normal or corrected-to-normal hearing. Each participant gave written informed consent before the experimental session. The study was approved by the New York University Abu Dhabi Institutional Review Board.

#### Stimuli and apparatus

The stimuli were presented on a 24-in. BenQ XL2411 monitor (1,920 × 1,080 pixels). Participants sat 57 cm from the monitor. The experiment was programmed in MATLAB using the Psychtoolbox extension (Brainard 1997; Kleiner et al. 2007; Pelli 1997). All visual stimuli were presented against a gray background. A black fixation cross (0.28° length) was displayed at the center of the screen throughout the whole experiment. The memory array consisted of four colored circles (with a radius of 0.69°) located on the corners of the imaginary square (with a radius of 3.33°), centered on fixation. On each trial, the colors of the circles were selected without replacement from a set of nine different colors (red, green, blue, yellow, orange, pink, purple, brown, and black). The valid retro-cue was a white arrow (with a length of 3.33° and width of 0.08°) with a head pointing to the location of one of the memory items. The neutral cue was composed of two white lines (6.67° length, 0.08° width) crossing at the fixation, with four endings pointing towards the four locations of the memory items. The memory test contained four circles occupying the same four locations as circles presented in the memory array. Three circles were gray with the white frames, and one circle was a colored probe item. The auditory stimuli were sinusoidal tones (300 and 900 Hz) presented bilaterally via headphones for 50 ms.

#### Design and procedure

**Fig. 1:**
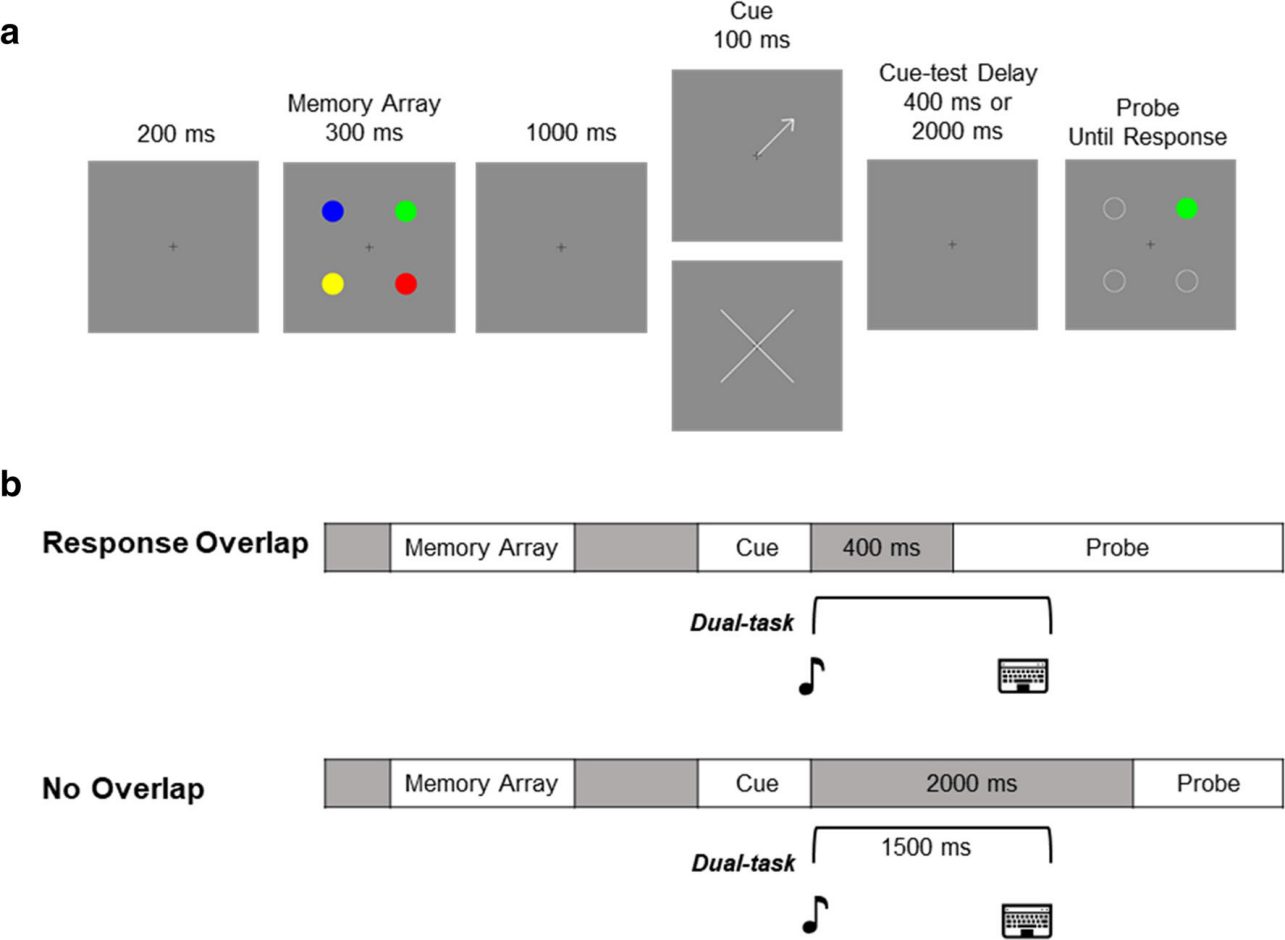
Schematic representation of experiment trials **a** and timing for different conditions **b**. Participants memorized a memory array (four items in Experiment 1, five items in Experiment 2) that was shown for 300 ms. After a 1,000-ms interval, the retro-cue was displayed for 100 ms: a valid cue indicated which item would be tested, whereas a neutral cue did not provide any information about the memory test. The retro-cue presentation was followed by a delay of 400 ms in the response overlap condition, or 2,000 ms in the no overlap condition. On single-task trials, no tone was presented during this delay. On dual-task trials, a tone stimulus was presented for 50 ms at the retro-cue offset, and participants made a speeded response to indicate whether the tone was high or low. For all trial types, after the memory delay, the change-detection probe was presented and remained on the screen until participants indicated whether the probe item matched the color of the item presented at the same location. While in Experiment 1 the articulatory suppression task was not included, in Experiment 2, participants were required to perform articulatory suppression (repeating the word cola) throughout every trial

Each trial (see Fig. 1) began with the presentation of the fixation cross for 200 ms. Afterward, the memory array was displayed for 300 ms, followed by a first delay period of 1,000 ms. Next, the retro-cue was presented for 100 ms. On *valid* trials, the retro-cue pointed towards the probed item with 100% validity. On *neutral* trials, the retro-cue provided no information on which item will be probed. The presentation of the memory test was preceded by a delay of either 400 ms on *response overlap* trials, or 2,000 ms, on *no overlap* trials, from the offset of the retro-cue. In the memory test, participants indicated whether the probed item was the same or different as the item presented at the same location in the memory array, via the left or right mouse clicks with their right hand. The memory display remained on the screen until an unspeeded response was made. Following the memory task, the feedback was provided by showing either “CORRECT” in green or “INCORRECT” in red for 1500 ms. The subsequent trial began after a 2000-ms delay, during which participants were shown a blank screen. On *single-task* trials, participants followed the procedure described above. On *dual-task* trials, the procedure was identical to that in *single-task* trials, except that participants also responded to a tone stimulus presented through the headphones at the retro-cue offset. Participants pressed the up-arrow key or the down-arrow key, in response to the 900-Hz or 300-Hz tones respectively, with their left hand. The responses had to be made as fast as possible. There were two dual-task conditions (across different sessions). In both *response overlap* and *no overlap conditions*, participants were instructed to respond to the tone task as quickly as possible and were required to respond to the tone task before providing a memory task response. On *response overlap* trials, there was only a short delay between tone task and memory test (400-ms cue-memory test delay). This meant that participants would be responding to the tone task well into the memory response stage, as in Janczyk and Berryhill (2014). On *no overlap* trials (2,000 cue-memory test delay), participants were forced to respond to the tone task before the memory probe was presented. Specifically, participants had 1,500 ms to respond to the tone task. When there was no response within this time limit, an alert sound (6,000 Hz) was presented for 100 ms, indicating to participants that they missed the chance to respond. Tone task performance feedback was presented simultaneously with memory task performance feedback (“Tone response correct” in green or “Tone response incorrect” in red).

Participants completed a total of 384 trials in a 90-min experiment divided into two sessions, *response overlap* and *no overlap* sessions, respectively. The order of the two sessions was counterbalanced across participants (by subject number). Participants completed a practice block of at least 20 *dual-task* trials before each session. Each session contained six 32-trial blocks, three *single-task* and three *dual-task*, in randomized order. In *single-task* blocks, the trials were evenly divided by the four conditions from 2 cue conditions (neutral, valid) × 2 memory task conditions (same, different). In *dual-task* blocks, trials were evenly divided by the eight trial types resulting from 2 tone stimuli (300 and 900 Hz) × 2 cue conditions × 2 memory task conditions.

### Results

Two participants were excluded from data analysis for failing to meet a priori cutoffs in performance: one because of chance-level performance (below 54.2%, the level at which there is 5% chance that the participant’s performance could be explained by random responses, determined by the 95% quantile of a binomial inverse cumulative distribution) on the memory task, and another because of low accuracy (below 75%) in the tone discrimination task.

#### Tone task

Tone task accuracy was high (96.2%). Tone task accuracy was analyzed with a 2 (cue condition: valid, neutral) × 2 (response: response overlap, no overlap) repeated-measures ANOVA. Performance was more accurate on neutral cue trials (96.8%) than on valid cue trials (95.5%), *F*(1, 33) = 9.94, *p* = .003, η_p_^2^ = .231, suggesting that performing the retro-cue task interferes with the tone response. Accuracy was also higher in the response overlap (97.4%) than in the no overlap condition (94.9%), *F*(1, 33) = 5.68, *p* = .023, η_p_^2^ = .147, presumably because there was a response deadline in the latter condition. The interaction between cue and response was not significant, *F*(1, 33) = 0.02, *p* = .894, η_p_^2^ = .001.

Mean correct RT for tone responses was 792 ms. Mean RTs were subjected to 2 (cue condition: valid, neutral) × 2 (response: response overlap, no overlap) repeated-measures ANOVA. We found longer RTs on valid cue trials (817 ms) than on neutral cue trials (766 ms), *F*(1, 33) = 13.74, *p* < .001, η_p_^2^ = .294. There was also a main effect of response overlap condition showing that participants responded more quickly in the no overlap condition (684 ms), where a response deadline is imposed, than in the response overlap condition (899 ms), *F*(1, 33) = 7.40, *p* = .010, η_p_^2^ = .183. The interaction between cue and response was not significant, *F*(1, 33) = 0.06, *p* = .816, η_p_^2^ = .002.

Overall, participants responded more quickly in the no overlap condition than in the response overlap condition. There are two potential causes of this. Perhaps the difference in RTs was due to the presence of the response deadline in the no overlap condition. However, speed was emphasized in both conditions, and the mean RTs in both conditions were well below 1,500 ms, regardless of whether there is a response deadline (1,500 ms). Therefore, we do not believe that the response deadline altered RTs. A more likely explanation is that responses were slowed in the overlap condition due to interference from the concurrent memory test.

#### Memory task

Tone RTs in the no overlap condition that were longer than 1,500 ms were excluded from the analysis of the memory task (since these RTs exceeded the response time limit). For all conditions, we excluded trials with incorrect tone responses. Lastly, trials with tone RTs above a cut-off value of 3 standard deviations from cell means were considered outliers, and they were also excluded from the analysis, leading to a data loss of 0.71% of the data points.

**Fig. 2:**
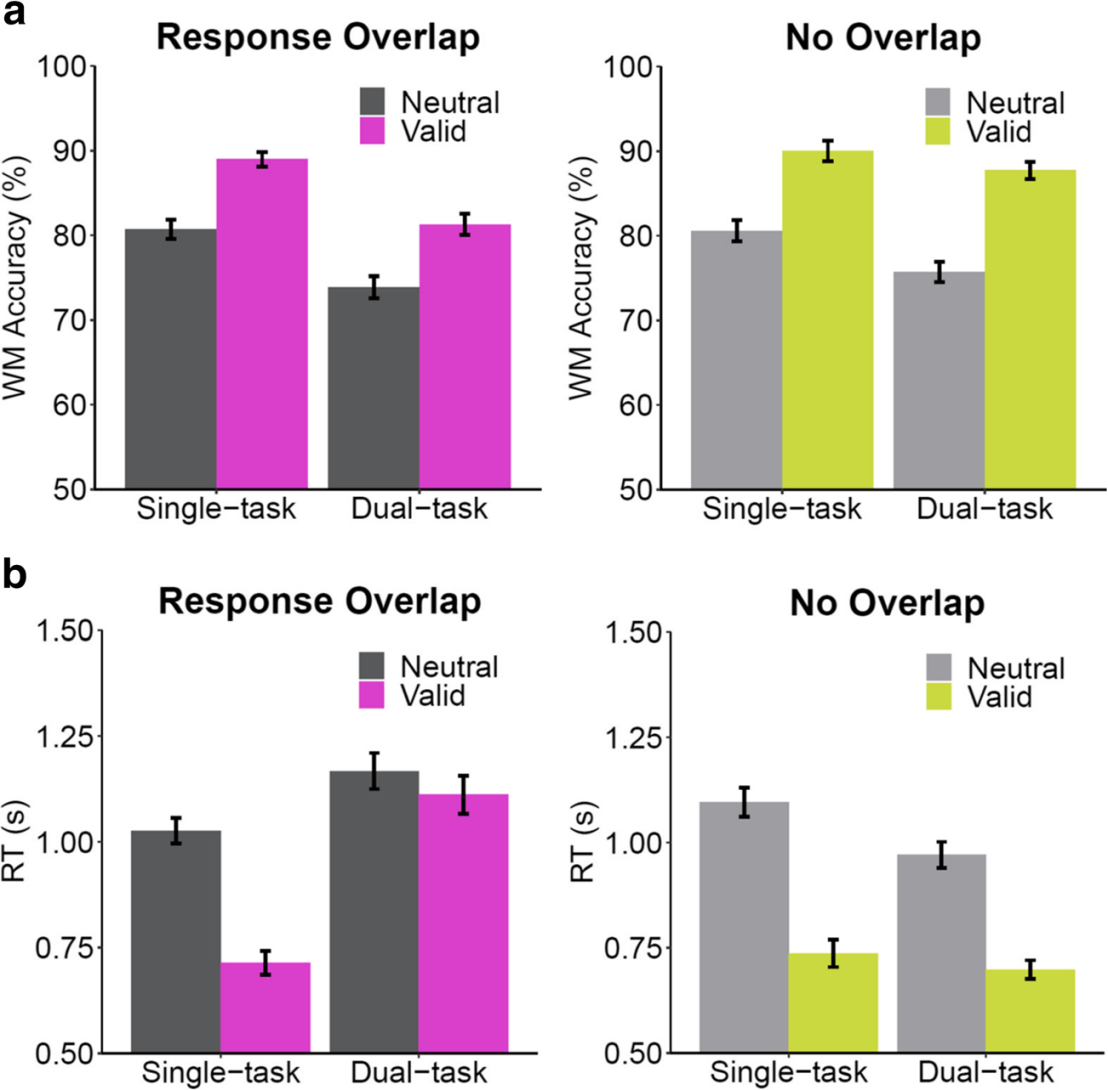
Mean change-detection accuracy ***a*** and mean memory response times (RTs) ***b*** for Experiment 1 as a function of cue (neutral, valid), task (single-task, dual-task) and response overlap conditions. Error bars represent standard errors of the mean

Memory task accuracy (Fig. 2a) was analyzed with a 2 (cue condition: valid, neutral) × 2 (task load: single-task, dual-task) × 2 (response: response overlap, no overlap) repeated-measures ANOVA. All main effects were significant. Performance was better in single-task (85.1%) versus dual-task (79.6%) conditions, *F*(1, 33) = 46.93*, p* < .001*,* η_p_^2^ = .587. Performance during valid cue trials (87.0%) was better than neutral cue trials (77.7%), *F*(1, 33) = 81.77*, p* < .001, η_p_^2^ = .712. Performance was better in no overlap (83.5%) compared to response overlap (81.2%), *F*(1, 33) = 4.22*, p* = .048*,* η_p_^2^ = .113. There was a significant interaction between cue condition and response overlap showing a smaller retro-cue effect (valid cue – neutral cue) in the overlap condition (7.9%) than in the no overlap condition (10.7%), *F*(1, 33) = 4.68, *p* = .038*,* η_p_^2^ = .124. There was also a significant interaction between task load and response overlap showing a smaller dual-task cost (single-task – dual-task) in the no overlap condition (3.6%) than in the overlap condition (7.3%), *F*(1, 33) = 14.99, *p* < .001, η_p_^2^ = .312, which does provide a hint that the cost of the attention task on memory depends on the amount of temporal overlap between the execution of this task and memory probe. The interaction between cue condition and task load was not significant, *F*(1, 33) = 0.34, *p* = .562, η_p_^2^ = .010, inconsistent with the claims of Janczyk and Berryhill (2014). The three-way interaction was not significant, *F*(1, 33) = 1.54, *p* = .224, η_p_^2^ = .044, failing to demonstrate that response overlap is mediating the cost of retro-cue effects from attentional load.

We further used the Bayesian model averaging (BMA) method (Hinne, Gronau, van den Bergh, & Wagenmakers, 2020) to weigh evidence for the interaction between cue condition and task load. This method weighs evidence for a particular effect across models that include the effect of interest against models that are stripped of the effect of interest. Specifically, the Bayes Factor (BF) here is a result of the sum of the prior probability of models with the effect of interest divided by the sum of the prior probability of models without the effect of interest. We found BF = 0.21, providing substantial evidence that the data are more probable under models without the two-way interaction between cue condition and task load than under the model with this interaction.

Memory task RTs (Fig. 2b) were analyzed with a 2 (cue condition: valid, neutral) × 2 (task load: single-task, dual-task) × 2 (response: response overlap, no overlap) repeated-measures ANOVA. Participants responded more quickly in single-task (893 ms) versus dual-task (987 ms) conditions, *F*(1, 33) = 7.55*, p* = .010*,* η_p_^2^ = .186. Participants also responded more quickly in valid cue trials (815 ms) than in neutral cue trials (1,065 ms), *F*(1, 33) = 113.51*, p* < .001, η_p_^2^ = .775. Memory RTs were faster in the no overlap condition (875 ms) compared to the response overlap condition (1,004 ms), *F*(1, 33) = 12.30*, p =* .001*,* η_p_^2^ = .272. The interaction between cue and task load was significant, *F*(1, 33) = 38.86, *p* < .001, η_p_^2^ = .541, showing a larger retro-cue effect (neutral cue RT – valid cue RT) in the single-task condition (335 ms) than in the dual-task condition (164 ms). There was a significant interaction between cue and response overlap showing a smaller retro-cue effect (neutral cue RT – valid cue RT) in response overlap (184 ms) compared to no overlap (316 ms), *F*(1, 33) = 30.69, *p* < .001, η_p_^2^ = .482. There was also a significant interaction between task load and response showing dual-task costs (dual-task RT – single-task RT) in the overlap condition (269 ms) but not in the no overlap condition (–82 ms), *F*(1, 33) = 56.89, *p* < .001, η_p_^2^ = .633. The three-way interaction was also significant, *F*(1, 33) = 13.52, *p* < .001, η_p_^2^ = .291. Paired *t*-tests revealed that the dual-task load decreased retro-cue effects in RT more in the response overlap (retro-cue effect in dual-task 56 ms vs. single task 312 ms), *t*(33) = 6.80, *p* < .001, than in the no overlap condition (retro-cue effect in dual-task 273 ms vs. single task 359 ms), *t*(33) = 2.52, *p* = .017.

Thus, the results in RTs replicate Janczyk and Berryhill’s finding that the retro-cue effect in RTs decreased under dual-task load. Perhaps valid retro-cues speed up memory responses by allowing for memory retrieval or evidence accumulation before the memory test onset (Shepherdson, Oberauer, & Souza, 2018; Souza et al., 2016), but the requirement to perform a secondary task would allow for less time to access the retro-cued item during the delay, thus reducing the retro-cue effects in RTs. Further, the three-way interaction revealed stronger effects of reduction in the overlap condition. This could reflect the fact that participants were still responding to the tone task when the memory test is presented, thereby preventing memory retrieval or shifts of attention towards the retro-cued item.

The retro-cue effect for accuracy was not modulated by attention (i.e., was not different between single- and dual-task conditions), but the retro-cue effects for RTs was reduced because of dual-task interference. Crucially, the reduction in RTs is larger in the response overlap condition, which is consistent with the idea that the close proximity of the tone and memory responses is important for observing the impaired retro-cue effects. Further, we observed greater dual-task costs (single-task – dual-task) in the overlap condition compared to the no overlap condition. This might mean that close temporal overlap between the responses leads to greater concurrence costs, suggesting that the response execution of the two tasks cannot be performed completely independently (Pashler, 1994).

## Experiment 2

Experiment 1 did not replicate Janczyk and Berryhill’s (2014) finding that the secondary tone task reduces the retro-cue effect in memory accuracy, even in the response overlapped condition. This is a surprising finding given that we used a nearly identical paradigm. However, there was one difference in the designs with the potential to explain the discrepancy. Janczyk and Berryhill’s task employed articulatory suppression. As visual information can be encoded both verbally and visually (Baddeley, 2000, 2012), studies investigating visual WM sometimes use articulatory suppression to restrict verbal encoding and rehearsal (Allen, Baddeley, & Hitch, 2006; Wheeler & Treisman, 2002). We did not include an articulatory suppression task in Experiment 1 given that recent studies have suggested that removing the possibility of verbal encoding does not impair performance in visual WM tasks (Morey & Cowan, 2004, 2005; Sense, Morey, Prince, Heathcote, & Morey, 2017), suggesting that either participants are not verbally encoding information or that verbalization of visual input does not impact performance. Nevertheless, it is possible that the lack of articulatory suppression in the current study leads to different results than those reported by Janczyk and Berryhill, either due to differences in how the information is encoded or the removal of additional attentional resources required by the articulatory suppression task. To more directly replicate the past work, we added an articulatory suppression task to Experiment 2. In addition, we increased the WM load to five items to make the memory task more difficult and thus to increase the likelihood of modulation of retro-cue effects by the requirement to execute the secondary tone task.

### Method

#### Participants

Forty-two New York University Abu Dhabi students and staff (21 females; mean age = 20.79 years; age range = 18–28 years) participated for course credit or subsistence of 50 AED per hour. The goal of this study was to collect 36 participants, consistent with the past study. We ended up with six additional participants due to human error. Importantly, qualitative conclusions did not change when the analysis was conducted without six additional participants. Thus, the analysis reported here includes data from all participants.

#### Design and procedure

The task was similar to Experiment 1, with the following modifications. Participants were asked to perform an articulatory suppression task by repeating the word “cola” aloud throughout each trial (Janczyk & Berryhill, 2014)**.** Moreover, the memory set was increased from four to five colored circles to increase task difficulty and likely increase the size of the retro-cue effect (Astle, Summerfield, Griffin, & Nobre, 2012; Gressmann & Janczyk, 2016; Souza et al., 2014).

### Results

Two participants were excluded because of chance-level memory accuracy.

#### Tone task

Mean tone accuracy was 94.3%. A 2 (cue condition: valid, neutral) × 2 (response: response overlap, no overlap) repeated-measures ANOVA showed that participants responded more accurately on neutral cue trials (95.5%) than on valid cue trials (93.1%), *F*(1, 39) = 26.96*, p* < .001, η_p_^2^ = .409. Accuracy was also higher in the response overlap (96.4%) than in the no overlap condition (92.2%), *F*(1, 39) = 32.70*, p* < .001, η_p_^2^ = .456. The interaction between cue and response was also significant, *F*(1, 39) = 4.93*, p* = .032, η_p_^2^ = .112, showing that the difference between neutral and valid cue conditions was larger in the no overlap (3.4%) than in the response overlap condition (1.5%).

Mean correct tone RT was 811 ms. A 2 (cue condition: valid, neutral) × 2 (response: response overlap, no overlap) repeated-measures ANOVA showed shorter RTs on neutral cue trials (788 ms) compared to the valid cue trials (834 ms), *F*(1, 39) = 10.17*, p* = .003, η_p_^2^ = .207. In addition, RTs were longer in the response overlap condition (928 ms) than in the no overlap condition (694 ms), *F*(1, 39) = 14.22*, p* < .001, η_p_^2^ = .267. The interaction between cue and response was not significant, *F*(1, 39) = 0.27*, p* = .605, η_p_^2^ = .007.

#### Memory task

**Fig. 3:**
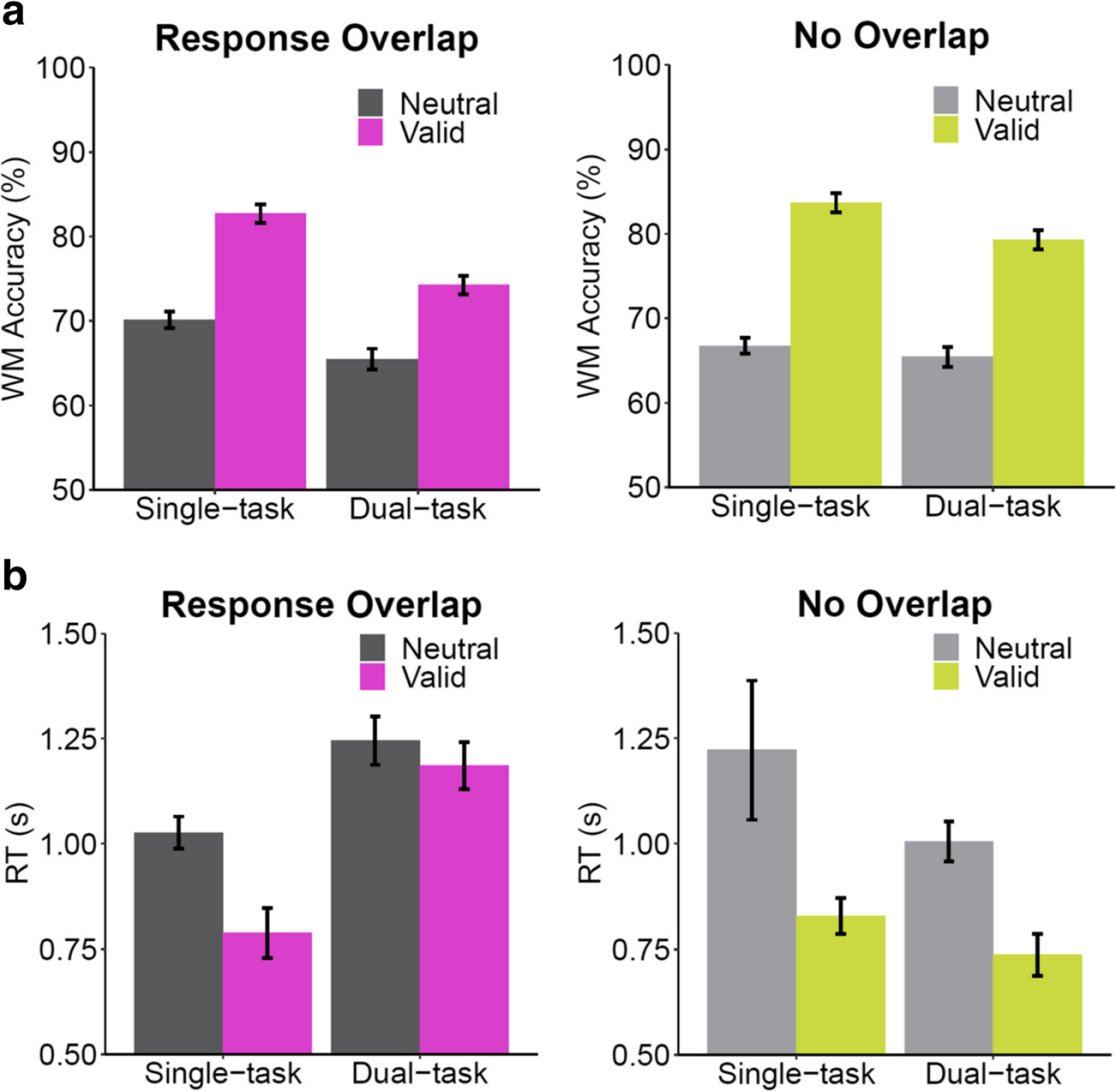
Mean change-detection accuracy **a** and mean memory response times (RTs) **b** for Experiment 2 as a function of cue (neutral, valid), task (single-task, dual-task), and response overlap conditions. Error bars represent standard errors of the mean

As in Experiment 1, trials with tone RTs larger than 1,500 ms and those with incorrect trial responses were first excluded from analysis of the memory task. We also excluded trials with tone RTs above a cut-off value of 3 standard deviations from cell means (0.64%). A 2 (cue condition: valid, neutral) × 2 (task load: single-task, dual-task) × 2 (response: response overlap, no overlap) repeated-measures ANOVA was performed on mean accuracy in the memory task (Fig. 3a). Valid cues improved memory accuracy (80.0%) compared to neutral cues (67.0%), *F*(1,39) = 212.29*, p* < .001, η_p_^2^ = .845. Performance was better in single-task trials (75.8%) compared to dual-task ones (71.1%), *F*(1,39) = 42.68*, p* < .001*,* η_p_^2^ = .523. There was no effect of response overlap, *F*(1,39) = 0.37*, p* = .545 *,* η_p_^2^ = .009, in contrast to Experiment 1.

With the modified design we replicated Janczyk and Berryhill’s finding of an interaction between cue and task load, *F*(1,39) = 7.30*, p* =.010, η_p_^2^ = .158. This reflects the fact that the retro-cue effect (valid cue – neutral cue) was higher in single-task (14.7%), relative to dual-task conditions (11.3%). As in Experiment 1, there was a significant interaction between cue and response showing a smaller retro-cue effect (valid cue – neutral cue) in the overlap condition (10.7%) than in the no overlap condition (15.4%), *F*(1, 39) = 10.35, *p* = .003*,* η_p_^2^ = .210. We also found an interaction effect between task load and response overlap showing a larger dual-task cost (single-task – dual-task) in the overlap condition (*M* = 6.5%) than in the no overlap condition (*M* = 2.9%), *F*(1,39) = 6.08, *p* = .018, η_p_^2^ = .135*.* The three-way interaction was not significant, *F*(1,39) = 0.15, *p* = .700, η_p_^2^ = .004*.*

We used the Bayesian model averaging (BMA) method (Hinne et al., 2020) to further weigh evidence for the three-way interaction across models that include the three-way interaction against models without the three-way intearaction. We found BF = 0.21, providing substantial evidence that the data are more probable under models without three-way interaction than under the model with three-way interaction.

Memory task RTs (Fig. 3b) were analyzed with a 2 (cue condition: valid, neutral) × 2 (task load: single-task, dual-task) × 2 (response: response overlap, no overlap) repeated-measures ANOVA. Participants responded more quickly in valid cue trials (885 ms) than neutral cue trials (1125ms), *F*(1,39) = 31.57*, p* < .001, η_p_^2^ = .447. In contrast to Experiment 1, there was no significant difference between single-task (967 ms) and dual-task trials (1,043 ms), *F*(1,39) = 1.62*, p* = .211*,* η_p_^2^ = .040. There was also no significant difference between response overlap (1,061 ms) and no overlap (948 ms) conditions, *F*(1,39) = 2.00*, p* = .165 *,* η_p_^2^ = .049. The interaction between cue and task load was marginally significant, *F*(1,39) = 3.84*, p* =.057, η_p_^2^ = .090, showing a larger retro-cue effect (neutral cue RT – valid cue RT) in single-task (315 ms) compared to dual-task trials (164 ms). There was a significant interaction between cue and response showing a smaller retro-cue effect in the overlap condition (149 ms) than in the no overlap condition (331 ms), *F*(1,39) = 5.09*, p* = .030 *,* η_p_^2^ = .115. We also found an interaction effect between task load and response overlap showing dual-task costs (dual-task RT – single-task RT) in the overlap condition (308 ms) but not in the no overlap condition (–154 ms), *F*(1,39) = 16.43, *p* < .001, η_p_^2^ = .296*.* The three-way interaction was not significant, *F*(1, 39) = 0.12, *p* = .736, η_p_^2^ = .003.

Thus, we found that the secondary task disrupts the retro-cue effect, but this disruption was not modulated by response overlap conditions. Even when comparing retro-cue effects (valid cue – neutral cue) in single- and dual-task conditions for response conditions separately, we found smaller retro-cue effects under dual-task load than single-task load in both the response overlap condition (*p* = .041) and the no overlap condition (*p* = .047).

To directly compare results from Experiments 1 and 2, we performed a mixed-design ANOVA on memory accuracy with experiment (Experiment 1 vs. Experiment 2) as between-subject factors and cue condition, task load, and response overlap as within-subject factors. Analyses showed that performance was better in Experiment 1 (82.4%) than in Experiment 2 (73.5%), where articulatory suppression was required, *F*(1, 72) = 24.06, p < .001, η_p_^2^ = .250. Performance was better in single-task (80.1%) versus dual-task (75.0%) conditions and for valid cue (83.1%) versus neutral cue (72.0%) conditions, (all *F*s > 89.81, all *p*s < .001). The significant interactions showed a smaller retro-cue effect (valid cue – neutral cue) in the response overlap (9.2%) versus no overlap (13.0%) condition, and a smaller dual-task cost (single-task – dual-task) in the no overlap (3.2%) versus response overlap (6.9%) condition, (all *F*s > 14.30 and all *p*s < .001). The effect of response overlap and the interaction between cue and task load was not significant (all *F*s < 3.66 and all *p*s > .060).

**Fig. 4:**
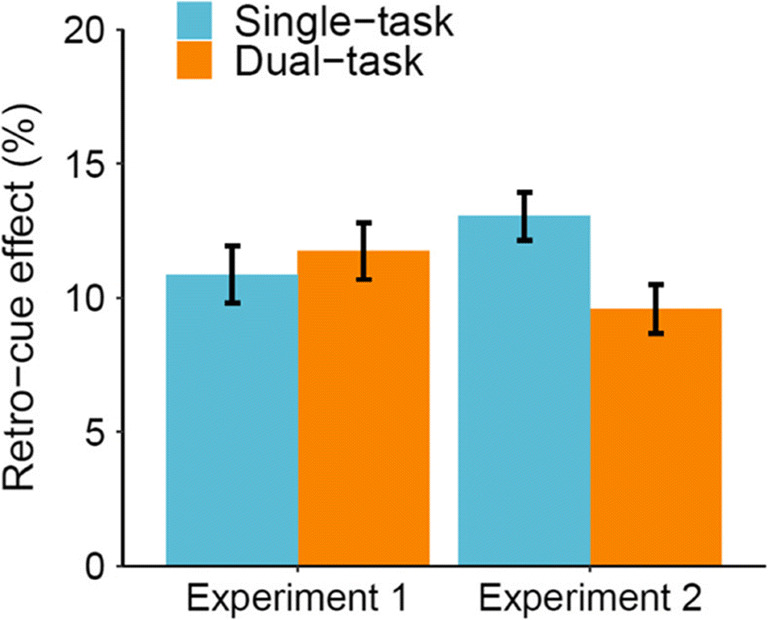
Retro-cue effect (valid cue – neutral cue) in memory accuracy for Experiments 1 and 2. Error bars represent standard errors of the mean

The interaction between cue condition and experiment was significant, *F*(1, 72) = 7.51, *p* = .008, η_p_^2^ = .094, showing a smaller retro-cue effect (valid cue – neutral cue) in Experiment 1 (9.3%) than in Experiment 2 (13.0%). Most critically, there was a significant interaction between experiment, cue condition, and task load, *F*(1, 72) = 4.90, *p* = .030, η_p_^2^ = .064, showing that the decrease in retro-cue effect under dual-task load was dependent on the experiment (Fig. 4). Specifically, in Experiment 1, the retro-cue effect (valid cue – neutral cue) was not reduced under the dual-task load (9.7%) compared to the single-task load (8.8%). However, in Experiment 2, the retro-cue effect (valid cue – neutral cue) was reduced under dual-task load (11.3%) compared to single-task load (14.7%). All other interactions were not significant (*F*s < 1.16, *p*s > .285). Taken together, the results of two experiments suggest that the retro-cue effect is sensitive to dual-task demands (replicating Janczyk & Berryhill, 2014), but this sensitivity seems to be dependent on the requirement to perform articulatory suppression.

## General discussion

Selection occurs both during perception and amongst items held in working memory. This raises the question of whether the mechanisms of selection are similar (Chun et al., 2011; Gazzaley & Nobre, 2012; Kiyonaga & Egner, 2013). In a recent study, Janczyk and Berryhill (2014) showed that the retro-cue effect (which is a measure of the benefit for a cued working memory item) decreased as a result of an attention-demanding task presented shortly after cue presentation. Based on these findings, the authors concluded that internal prioritization induced by the retro-cue requires attention. This result stands in contrast to the findings from other researchers that failed to observe any reduction of retro-cue effect because of the requirement to perform the secondary task (Hollingworth & Maxcey-Richard, 2013; Makovski & Pertzov, 2015; Rerko et al., 2014).

There are differences in tasks and methods that could explain the differences in findings across studies (e.g., only Janczyk & Berryhill, 2014, presented the secondary task in close temporal proximity to the retro-cue). Here we explored whether the findings by Janczyk and Berryhill could have an alternative explanation. Specifically, in their paradigm, the secondary tone task response overlapped with the memory test response. Thus, it is difficult to infer whether the impaired retro-cue effects arise from secondary task interference with the selection process in memory or disruption of other processes related to the memory response.

We used a paradigm similar to their study, but crucially we included a condition with a longer delay between the tone task and the memory test. Furthermore, we required participants to respond to the tone task before the memory task to prevent responses for the two tasks from overlapping with each other. If the retro-cue effect is not reduced when tone responses are completed before the memory test is shown, it would suggest that the results Janczyk and Berryhill obtained are not due to secondary task interference with internal prioritization. It could instead mean that the processing of the two tasks together prevents the memory representation from being accessed or retrieved until the tone task is completed, which could disrupt the effect of the retro-cue, particularly if the retro-cue acts to prioritize items during response (Astle et al., 2012). The results showed that WM performance was improved by the retro-cue, but was broadly impaired by the presence of the secondary tone task. Although we did not present tones in the single-task condition, we reasoned that including tones in both the single-task and dual-task conditions would not lead to different results based on findings reported by Experiment 2 in Janczyk and Berryhill. In this experiment, they found that the retro-cue effect was reduced when the tone task was presented close to the retro-cue compared to when it was presented long before the cue. These results suggest that the reduction in retro-cue effects was not due to the mere presence of tones in the dual-task condition. In line with previous studies (Magen, 2017; Oberauer, 2018), we also found a higher dual-task cost on trials with a shorter interval between the tone presentation and the memory test screen. This finding may suggest that attention is involved in memory retrieval. That is, the demands of the tone task may have delayed or impaired the memory response when there was overlap between the two tasks. In addition, we found a larger retro-cue effect at longer cue-test delays, supporting the view that the retro-cue effect requires time to develop (Pertzov et al., 2013; Souza et al., 2014, 2016; Wallis et al., 2015).

Most critically, Experiment 1 did not replicate the key finding of Janczyk and Berryhill (2014). We did not find a decrease in the retro-cue effect under dual-task interference for memory accuracy, though we did observe a decrease in the retro-cue effects for RTs. The results in RTs might suggest that the secondary task prevented evidence accumulation for the retro-cued item (Shepherdson et al., 2018; Souza et al., 2016). However, this study was not an exact replication of Janczyk and Berryhill. Most critically, we left out the articulatory suppression task. To explore whether this is critical to replicate the findings, in Experiment 2 we included the same articulatory suppression task used in their study. When articulatory suppression was included, we found that the retro-cue effect in accuracy was reduced by dual-task demands, and we observed a marginal trend in RTs in the same direction. This finding suggests that the reduction of retro-cue effects observed in the current study and in the study by Janczyk and Berryhill did not solely depend on secondary task interference occurring close to the retro-cue, but also required a concurrent articulatory suppression task. Lastly, in Experiment 2, we found no evidence for an interaction between cue condition, task load, and response overlap, suggesting that the observed impairment in the retro-cue effect was independent of the temporal proximity of the secondary task to the memory task, in contrast to our predictions.

While we found similar results to Janczyk and Berryhill (2014) in Experiment 2, the failure to find any costs of the tone task on retro-cueing in Experiment 1 places important limitations on when these findings are likely to be observed. One intuitive explanation of why dual-task interference resulted in the reduction of retro-cue effects only when articulatory suppression was present is that visual WM representations were strengthened by verbal encoding or/and rehearsal (Brown, Forbes, & McConnell, 2006; Dent & Smyth, 2005; Postle, D’Esposito, & Corkin, 2005; Postle & Hamidi, 2007), which may have reduced the harmful impact of the interference from the tone task. However, this explanation has some weak points. A considerable number of studies showed no evidence for improvement of memory performance when there is an opportunity to verbally encode or rehearse visual information (Mate, Allen, & Baqués, 2012; Morey & Cowan, 2004, 2005). Also, a recent study by Sense et al. (2017) using a more comprehensive analysis (both descriptive analysis and Bayesian state-trace analysis) showed that articulatory suppression had no effect on change-detection performance. Lastly, some previous studies that showed no reduction in the retro-cue effect because of distraction also required participants to perform articulatory suppression (Hollingworth & Maxcey-Richard, 2013; Makovski & Jiang, 2007). These findings suggest that the presence of the articulatory suppression alone may not account for the disparity in findings.

Another possible explanation is that the presence of articulatory suppression imposes additional demands, which, in combination with the tone task, result in even greater task demands. Although both articulatory suppression and the tone discrimination task can be considered as simple tasks, the requirement to execute these tasks simultaneously may consume more resources (Baddeley, Chincotta, & Adlam, 2001; Bryck & Mayr, 2005; Emerson & Miyake, 2003; Garavan, 1998; Janczyk & Grabowski, 2011; Janczyk, Wienrich, & Kunde, 2008; Kirkham, Breeze, & Marj-Beffa, 2012; Miyake, Emerson, Padilla, & Ahn, 2004; Saeki & Saito, 2004). In other words, it is possible that the process of internal prioritization by the cue can be severely disturbed only when the interference is strong, and such strong interference can arise from the requirement to execute multiple tasks at the same time. The interesting question is what type of task or combination of tasks impose enough interference to disturb the process of internal prioritization. The secondary tone task that Janczyk and Berryhill (2014) and the current study employed is specifically thought to disrupt central attention. Other studies, which presented secondary attention tasks long after the retro-cue offset, found that the retro-cue effect is resistant to distraction of central attention (e.g., visual and auditory digit classification tasks; Makovski & Pertzov, 2015) or visual attention (e.g., visual search task; Hollingworth & Maxcey-Richard, 2013; color classification task; Rerko et al., 2014). Future work can further investigate the attentional requirements of the retro-cue effect and the role of subvocal rehearsal by examining the addition of general task loads that are not specifically involved in preventing verbal rehearsal.

How might the tone task be disrupting prioritization? There are multiple theories that differ on what mechanisms are behind the retro-cue effect (for a review, see Souza & Oberauer, 2016). Prominent hypotheses include: that retro-cues protect WM representations from time-based decay (Matsukura, Luck, & Vecera, 2007; Pertzov et al., 2013), that retro-cues guide refreshing in WM (Rerko & Oberauer, 2013; Souza, Rerko, & Oberauer, 2015), that retro-cues lead to the removal of no longer relevant items from WM (Kuo, Stokes, & Nobre, 2012; Souza et al., 2014), that retro-cues give more time for evidence accumulation, which may lead to better memory performance (Souza et al., 2016), or that retro-cues protect task-relevant items in WM from perceptual interference (Landman et al., 2003; Makovski & Jiang, 2007; Makovski, Sussman, & Jiang, 2008; Matsukura et al., 2007; Souza et al., 2016). These hypotheses are not necessarily mutually exclusive, and they may combine to explain how retro-cued information is protected from being lost from memory. Taxing attention with a tone task could prevent or reduce the effectiveness of any of these processes. For example, it is proposed that attentional refreshing or strengthening (Souza et al., 2015; Souza & Oberauer, 2016) requires attention (Camos et al., 2018; Raye, Johnson, Mitchell, Greene, & Johnson, 2007; Souza & Oberauer, 2017) and thus may be susceptible to interference from our tone task. Future work is necessary to fully explore how a tone task might disrupt these processes.

Rather than impairing the mechanism of selection or prioritization in retro-cueing, another possibility is that the disruption of retro-cue effects may occur simply because the higher load increases the probability that the *wrong item* is receiving the benefits of selection. For example, perhaps the attentional demands of the tone task and articulatory task lead to an increased probability that an incorrect item is tagged as relevant. An increase of such swaps during the tone task would lead to smaller retro-cue effects.

Taken together, our results not only provide further evidence for a role of attention in the setting up of a retro-cue, but suggest limitations on this interference. The current study demonstrated that the retro-cue effects decreased only when there was the requirement to perform articulatory suppression in addition to the secondary tone task. In addition, the study verified that the impairment of the retro-cue effects is not dependent on the interaction between the secondary tone task response and the memory probe. Thus, our findings confirm that prioritization of information in WM is sensitive to disruption from processing involved in multitasking situations and is not automatic or free from attentional demands.
